# Strategies for data acquisition using ultrasonic phased arrays

**DOI:** 10.1098/rspa.2018.0451

**Published:** 2018-10-17

**Authors:** A. Velichko, A. J. Croxford

**Affiliations:** Department of Mechanical Engineering, University of Bristol, Queens Building, University Walk, Bristol BS8 1TR, UK

**Keywords:** ultrasonic arrays, data acquisition, plane wave imaging

## Abstract

Ultrasonic phased arrays have produced major benefits in a range of fields, from medical imaging to non-destructive evaluation. The maximum information, which can be measured by an array, corresponds to the Full Matrix Capture (FMC) data acquisition technique and contains all possible combinations of transmitter–receiver signals. However, this method is not fast enough for some applications and can result in a very large volume of data. In this paper, the problem of optimal array data acquisition strategy is considered, that is, how to make the minimum number of array measurements without loss of information. The main result is that under the single scattering assumption the FMC dataset has a specific sparse structure, and this property can be used to design an optimal data acquisition method. An analytical relationship between the minimum number of array firings, maximum steering angle and signal-to-noise ratio is derived, and validated experimentally. An important conclusion is that the optimal number of emissions decreases when the angular aperture of the array increases. It is also shown that plane wave imaging data are equivalent to the FMC dataset, but requires up to an order of magnitude fewer array firings.

## 1. Introduction

Ultrasonic arrays are now routinely used in industry for non-destructive testing and assessment of the structural integrity of materials. Modern computational power makes it possible to collect raw array data first and then perform image reconstruction in post-processing. One common data collection method, which is now available in many commercial ultrasonic array systems is Full Matrix Capture (FMC) [1–4]. In this case, the ultrasonic wave is consecutively transmitted by each array element and the reflected signal is measured by all array elements simultaneously. The advantage of this method of array data acquisition is that an FMC dataset contains the maximum possible information that can be measured by an array from a given position. It means that data corresponding to any array acquisition method can be synthesized from the FMC dataset and various imaging techniques can be applied [1,5–10]. Moreover, advanced signal processing becomes possible. For example, scattering matrices of defects can be extracted and used for their characterization [11–15].

However, the FMC data acquisition method also has some disadvantages. The first important issue is related to the volume of measured array data. If an ultrasonic array has *N* elements, then the corresponding FMC dataset consists of *N*^2^ time signals. In many industrial cases, an inspection is carried out by physically scanning a phased array to cover a large area. Although the data volume for a single FMC set is relatively small, the total amount of inspection data in this case can be extremely large, containing terabytes of data.

Secondly, in order to measure the FMC data *N* transmissions are required, which can take too long for some applications. Note, that the technological advances in the last few years have made it possible to perform ultrasonic imaging using the FMC data acquisition method in near ‘real time’, where this means frame rate of tens frames per second [16]. This is close to the fundamental physical limit, which is related to the finite speed of ultrasonic wave propagation and their decay in materials. Although this imaging rate is fast enough in some industrial cases, there are multiple applications which require a much faster data acquisition rate of several hundred (or, in some cases, even thousands) frames per second. Regarding industrial non-destructive testing applications, well-known examples are pipeline inspection using pigging [17] and railway track inspection [18]. In these cases, the inspection vehicle moves at tens of metres per second so an ultrasonic frame rate of 10 frames per second is wholly insufficient. In addition, a very high frame rate opens the possibility for many other applications, such as real-time monitoring of fatigue crack growth.

One possible solution to reduce the amount of measured data is based on compressive sensing theory [19], which provides a method to reconstruct data from far fewer samples than required by Nyquist sampling theorem. The main condition under which this reconstruction is possible is that the data are sparse. In the application of this theory to ultrasonic imaging the data to be reconstructed represents an image, and sparsity is usually interpreted as a small number of scatterers present in the material [20,21]. This approach is not generalizable and lacks a formal definition to assess its performance in different inspections.

The problem of reduction of the number of ultrasonic array firings has been intensively studied by many researchers. The common source method uses all elements to transmit simultaneously, so that a plane wave propagates into the structure and an image is created from the scattered waves [22–25]. Another possibility is to use just a few array elements as transmitters in order to produce the best image for a given number of firings [26,27]. However, in both cases the reduction in the data acquisition time comes at the expense of significantly reduced image quality. Alternatively, iterative imaging methods have been suggested in order to suppress the aliasing noise [28,29]. These techniques help to reduce imaging artefacts, but the signal processing time increases in proportion to the number of iterations. The main progress in this area has been achieved in the medical imaging field using plane wave excitations [30,31]. It has been shown that it is possible to achieve good quality imaging with a relatively small number of transmitted plane waves. The image quality in plane wave imaging is affected by a number of parameters, specifically the number of transmitted plane waves and the maximum steering angle. A method for optimizing these parameters based on Pareto optimality has been developed in [32]. It has also been demonstrated that plane wave imaging techniques give good results in non-destructive testing applications with much smaller number of emissions compared to the FMC method [33,34]. However, the physical reason for this has not been explained. Also, it remains unclear if there is a fundamental relationship between maximum steering angle and the number of plane wave transmissions.

The key question investigated in this paper is how to optimize the array data acquisition process so it measures the same amount of information as the FMC method, but using fewer measurements. The crucial observation is that under the single scattering assumption an FMC dataset has a specific sparse structure, which is independent of the number of scatterers. This property can be used to construct the optimal array data measurement procedure. It is shown that plane wave imaging represents one possible implementation of the optimal data acquisition approach. Also, the angular sampling criterion, which relates the number of plane wave to the maximum steering angle, is derived. The proposed plane wave sampling theory is experimentally validated on an aluminium sample with volumetric defects. Finally, the key applications of this approach to a range of fields are discussed.

## 2. Structure of ultrasonic array data

### (a) Formulation of the problem

**Figure 1.**
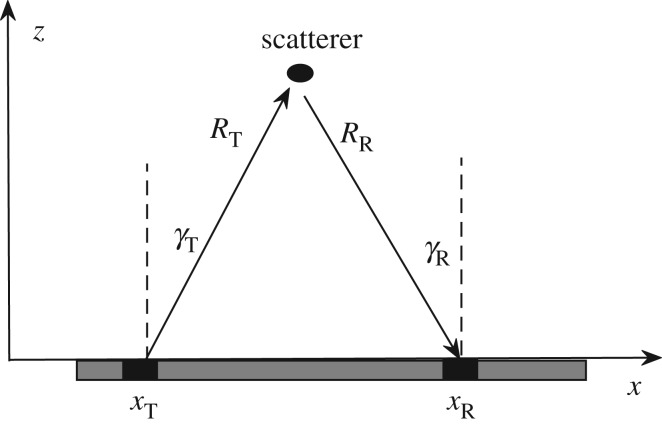
Array measurement geometry.

In this paper, for simplicity, two-dimensional imaging using a one-dimensional linear array is considered. However, all results can be extended to three-dimensional imaging using a two-dimensional array. The system is illustrated schematically in figure 1. A two-dimensional elastic isotropic half-space is considered with Cartesian coordinate axes (*x*, *z*) defined with the *z*-axis normal to the surface of the half-space. In the simulations shown below the material is aluminium (Young's modulus: 69 GPa, Poisson's ratio: 0.334, density: 2700 kg m^−3^). A 5 MHz, 64 element array is used as an example throughout this paper, and the element pitch of the array is 0.6 mm (i.e. 0.5*λ* at the centre frequency).

**Figure 2.**
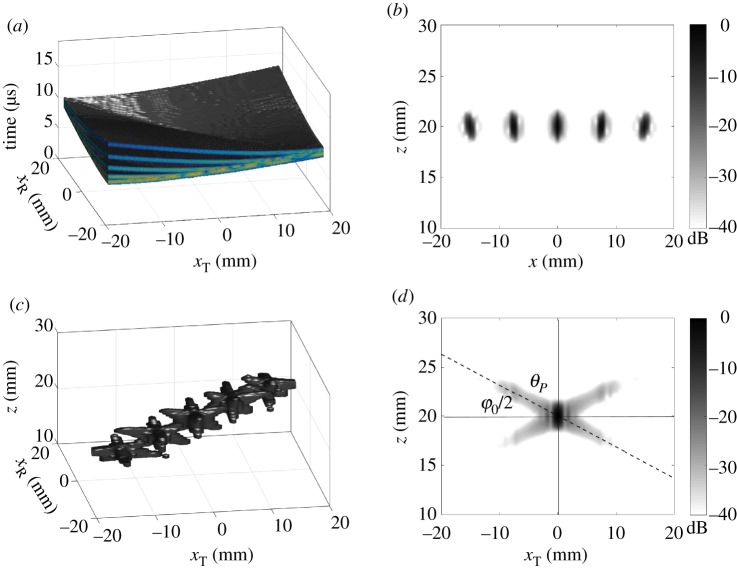
Modelling results for five point scatterers: (*a*) FMC data, −40 dB isosurface image; (*b*) back-propagation two-dimensional image; (*c*) generalized back-propagation image showing −40 dB isosurfaces; (*d*) slice of the generalized image at *x*_R_ = 0. Dashed line indicates the direction of side-lobes (angle *θ*_*P*_) given by (2.7), angle *φ*_0_ is the array half angular aperture.

In general, the ultrasonic array data, *g*, can be measured in many different ways, for example, by applying various time-delays to the array elements or using different subsets of array elements in transmission and reception. However, the maximum possible amount of information that can be collected by the array in a particular position is contained in the full matrix of transmitter–receiver array data, measured using the FMC technique. The FMC data depend on three variables: position of transmitter array element, *x*_T_, position of receiver array element, *x*_R_ and time, *t*, and can be visualized as a three-dimensional cube of data, *g*_fmc_(*t*, *x*_T_, *x*_R_) (figure 2*a*). One example of an alternative array data capture technique is the plane wave imaging method [30,33]. In this case, the measured array data are given by a dataset *g*_pw_(*t*, *γ*_T_, *x*_R_), which depends on the angle of transmitted plane wave, *γ*_T_, position of receiver array element, *x*_R_ and time, *t*.

The main question, investigated in this paper, is if there is an optimal array data measurement technique, which allows the same amount of information to be collected as FMC, but using a smaller number of measurements. Mathematically, it means, that the data *g* has a smaller number of components, than the FMC data, *g*_fmc_, and there exists a one-to-one relationship between them:

2.1 $$g_{\mathrm{fmc}}=T[g]\;\mathrm{and}\;g=T^{-1}[g_{\mathrm{fmc}}],$$

where *T* is the mapping operator from the new measurement space *g* into the FMC measurement space *g*_fmc_.

Expression (2.1) means that measurements *g*_fmc_ and *g* are equivalent to each other. One important practical implication is that in this case all data processing methods available for the FMC data, *g*_fmc_, can also be applied to the data *g*. For example, scattering matrices for different defects can be extracted from the raw measured data and then used for defect characterization [15]. Another important observation from relationship (2.1) is, that if the number of measurements in *g* is smaller than the dimension of the FMC data *g*_fmc_, then different measurements in the FMC dataset are not independent. Therefore, the main goal of this paper is to find possible dependencies in the FMC data and then use them to design the optimal measurement procedure. It is important to note that these sparsity relationships will be based on fundamental properties of the FMC data and array and not rely on the assumption of a small number of scatterers or other qualitative comparisons.

### (b) Concept of imaging as a data compression method

Conventionally, the amount of information in the array datasets *g*_fmc_ and *g*, is assessed by comparing the ultrasonic images, *I*_fmc_ and *I*, obtained using data *g*_fmc_ and *g*, respectively. If *L*_fmc_ and *L* are imaging operators for the *g*_fmc_ and *g* datasets, then

2.2 $$I_{\mathrm{fmc}}=L_{\mathrm{fmc}}[g_{\mathrm{fmc}}],\;I=L[g].$$

Traditionally, it was assumed, that if images have similar quality (for example, based on the signal-to-noise ratio or contrast metrics), then there is no loss of information. However, in general, the equality *I*_fmc_ = *I* does not necessarily imply that the data *g*_fmc_ and *g* are equivalent in the sense of relationship (2.1), key if more advanced processing is to be carried out. A sufficient condition for the existence of a mapping operator *T* between two datasets is that the corresponding imaging operators are reversible. In this case,

2.3 $$T=L_{\mathrm{fmc}}^{-1}L\;\mathrm{and}\;T^{-1}=L^{-1}L_{\mathrm{fmc}}.$$

The construction of an inverse imaging operator requires the range of the imaging operator to be known, which in turn requires a specific model of the scattering mechanism. For example, if scatterers are considered as a collection of frequency-independent point scatterers, then an image represents a reconstruction of the scatterer distribution function. In this case, the image-to-data operator is represented by the forward model of wave scattering from a known distribution of point scatterers. Alternatively, the imaging operator *L*_fmc_ can be considered globally [12], as an operator in the vector space *g*_fmc_. The advantage of this approach is that the reversibility of the imaging operator becomes model-independent. For example, it was shown that the back-propagation imaging method is reversible in such a global sense [12].

The analysis in this paper is based on the assumption that multiple scattering between different scatterers is small compared with the single scattering contribution, so a single scattering model is valid. It is important to stress that no other assumptions about the physical nature of scattering are made.

The modelling example of FMC data for five-point scatterers, located at a depth of 20 mm from the array is shown in figure 2*a*. It can be seen that the scattering data are spread over all combinations of transmit–receive signals, and it is difficult to see any general dependencies between different components of the FMC dataset. However, the main characteristic of the single scattering data are that for every scatterer all scattered signals come from a single location, corresponding to the position of the scatterer. The majority of the array imaging methods are based on this property and effectively focus the scattered signals back at the scatterer location. If the imaging method is reversible, then it is possible to convert the image back to the array data. Therefore, in this case the image represents an alternative form of the array data. Moreover, because scattered signals from different scatterers are localized in the imaging space, the imaging algorithm can be considered as an array data compression operation.

### (c) Reversible back-propagation imaging method

In this section, the analysis of the reversible back-propagation imaging operator is performed which helps to reveal data redundancies in the FMC dataset.

In general, the transmitter and receiver elements are designed to be sensitive to the longitudinal wave mode only, so shear waves and mode conversion effects are not considered. The back-propagation imaging method can be represented by a linear operator, *B*, which converts FMC data, *g*_fmc_(*t*, *x*_T_, *x*_R_), into the generalized image, *b*(*z*, *x*_T_, *x*_R_) [12],

2.4 $$b(z,x_{T},x_{R})=B[g_{\mathrm{fmc}}(t,x_{T},x_{R})],\;B=F^{-1}HF.$$

Here *F* is a two-dimensional Fourier transform with respect to the spatial coordinates *x*_T_, *x*_R_, *F*^−1^ is the inverse Fourier transform and *H* is the back-propagation of the angular spectrum operator. Note that the physical meaning of the generalized image, *b*(*z*, *x*_T_, *x*_R_), is transmitter–receiver array data, measured at time *t* = 0 by an array located at depth *z* [12]. Alternatively, it can be considered as beamforming with different transmit, (*x*_T_, *z*), and receive, (*x*_R_, *z*), focusing.

The operator *H* converts the time data into a function of propagation distance and can be written in the form of a one-dimensional Fourier transform (see appendix Aa). Therefore, each operator in expression (2.4) for the back-propagation operator *B* is reversible and the generalized image *b*(*z*, *x*_T_, *x*_R_) can be converted back into the array data *g*_fmc_(*t*, *x*_T_, *x*_R_):

2.5 $$g_{\mathrm{fmc}}(t,x_{T},x_{R})=B^{-1}[b(z,x_{T},x_{R})],\;B^{-1}=F^{-1}H^{-1}F.$$

where *B*^−1^ is the inverse imaging operator.

The conventional two-dimensional image of scatterer position *I*(*x*, *z*) is given by the pulse-echo data *x*_T_ = *x*_R_ of the generalized image

2.6 I(x,z)=b(z,x,x)].

Therefore, the generalized image *b*(*z*, *x*_T_, *x*_R_) contains more information than is necessary for localization of the scatterers. However, the extra information corresponding to the non-diagonal data *x*_T_≠*x*_R_ is crucial for the inverse imaging [12].

### (d) Point spread function in the generalized image domain

Figure 2*b* shows the conventional two-dimensional back-propagation image, i.e. in the pulse echo *x*_T_ = *x*_R_ plane, for the modelling example of five-point scatterers. The corresponding generalized image is presented in figure 2*c*. It is seen that the back-propagation operator focuses the energy from each scatterer into the vicinity of its location, and, therefore, the data in the generalized image domain are localized around this pulse-echo plane *x*_T_ = *x*_R_. Note that the generalized image can be considered as a different representation of the original FMC dataset shown in figure 2*a*, as they can be transformed between the two representations using the back-propagation and inverse back-propagation operators. Comparison of figure 2*a*,*c* reveals the fundamental sparse nature of the FMC dataset. It is clear that the degree of sparseness is defined by the point spread function (PSF) of the array in the generalized image domain. The structure of the PSF is investigated in this section.

For simplicity of the initial analysis, it is assumed that the array elements are omnidirectional and a point scatterer is located at depth *z*_0_ directly below the geometrical centre of the array (*x*_0_ = 0). An example of the PSF in the generalized image domain is shown in figure 2*d*. The amplitude of the side lobes can be estimated analytically using asymptotic analysis of the back-propagation imaging operator. The detailed derivation is given in appendix Ab, and only final results are summarized below.

The main side lobes are located in the (*x*_T_, *z*), (*x*_R_, *z*) planes in the directions *θ* = *θ*_*P*_ (figure 2*d*):

2.7 $$\theta_{P}=\frac{\pi }{2}\mp \frac{\varphi_{0}}{2},$$

where the angle *θ* is an elevation angle in the (*x*_T_ − *x*_0_, *x*_R_ − *x*_0_, *z* − *z*_0_) space, and *θ* = 0 corresponds to the direction of the positive *z*-axis. The angle *φ*_0_ corresponds to the maximum scattered angle measured by the array (half angular aperture of the array),

2.8 $$\tan\varphi_{0}=\frac{L}{2z_{0}},$$

where *L* is the array aperture.

The quantity of interest is the relative side lobe level as a function of distance *r* and array angular aperture *φ*_0_:

2.9 $$\delta_{P}(r,\varphi_{0})=\frac{P(r,\theta_{P})}{P(0,0)},$$

where *P*(*r*, *θ*) is the point spread function and *r* is the radial distance in the (*x*_T_ − *x*_0_, *x*_R_ − *x*_0_, *z* − *z*_0_) space with the centre at the location of the scatterer.

It is convenient to introduce dimensionless distance $\tilde{r}=r/\lambda$, where *λ* is the ultrasonic wavelength at the centre frequency *f*_0_. Then the asymptotic behaviour of the PSF in the far field $\tilde{r}\gg 1$ is given by

— if $\tilde{r}\varphi_{0}^{3}\ll 1$
  2.10 $$\delta_{P}(\tilde{r},\varphi_{0})=\frac{1}{4\pi \tilde{r}}\;\frac{1}{\varphi_{0}\cos(\varphi_{0}/2)},$$
— if $\tilde{r}\varphi_{0}^{3}\geq 1$
  2.11 $$\delta_{P}(\tilde{r},\varphi_{0})=\frac{1}{8\pi {\tilde{r}}^{3/2}}\;\frac{1}{\varphi_{0}^{2}\cos(\varphi_{0}/2)\sqrt{\sin(\varphi_{0}/2)}}.$$

Qualitatively, expression (2.10) is applicable when the half array angular aperture *φ*_0_ is relatively small. This corresponds to the situations when the array size is small or the scatterer is located far from the array. On the other hand, when the array angular aperture *φ*_0_ increases, the array focusing capability becomes better. Consequently, the decay rate of the PSF also increases according to expression (2.11).

Figure 2*d* shows the *x*_T_ − *z* plane (*x*_R_ = 0) of the generalized image of the PSF for the scatterer located in the centre of the array. The dashed line corresponds to the theoretically predicted side-lobe direction. Note that the half angular aperture of the full array is 40°. However, large angles contribute mainly to the noise and, therefore, an additional angular filter of *φ*_0_ = 35° was applied in the wavenumber domain. In this case, according to expressions (2.7), (2.11) the side-lobes direction is *θ*_*P*_ = 72.5° and the side-lobes length at −40 dB in *x*_T_ direction is 9 mm. It can be seen that these theoretical estimations are in a good agreement with figure 2*d*.

## 3. Optimal data acquisition strategies

### (a) General approach

In this section, the sparse nature of the FMC dataset in the generalized image domain is used to design the optimal data acquisition procedure. For the analysis, it is convenient to rewrite the back-propagation imaging operator (2.4) as an operator acting on the angular spectrum *G*(*t*, *k*_*x*(*T*)_, *k*_*x*(*R*)_) = *F*[*g*_fmc_(*t*, *x*_T_, *x*_R_)]:

3.1 $$\begin{array}{rl} & b(z,x_{T},x_{R})=F^{-1}H[G(t,k_{x(T)},k_{x(R)})]\\ \;\mathrm{and}\; & G(t,k_{x(T)},k_{x(R)})=H^{-1}F[b(z,x_{T},x_{R})]. \end{array}\}$$

Note, that the Fourier transform operator *F* acts on the spatial coordinates *x*_T_, *x*_R_, and the back-propagation of angular spectrum operator *H* acts on the variable *z* only.

If the array data are collected using the FMC technique, then the sampling in the wavenumber domain (*k*_*x*(*T*)_, *k*_*x*(*R*)_) is defined by the array aperture *L* as

3.2 $$\Delta k_{x(T)},\;\Delta k_{x(R)}=\frac{2\pi }{L}.$$

In the previous section, it was shown that the generalized image *b*(*z*, *x*_T_, *x*_R_) has a specific sparsity, when all data are localized around the pulse-echo plane *x*_T_ = *x*_R_. This means that the sampling rule (3.2) is too conservative and results in oversampling. The concept of the optimal data acquisition method is to design the optimal sampling in the wavenumber domain, so that it reflects the sparse property of the generalized image.

From the practical point of view, an important question is how to implement the sampling scheme in the wavenumber domain using array measurements. There are multiple possible sampling schemes which are consistent with the sparsity of the generalized image, however, not all of them allow practical implementation using conventional ultrasonic array instrumentation. Therefore, in this paper an additional optimization criterion of minimal number of array firings is used. In this case, it is convenient to represent the Fourier transform operator as *F* = *F*_T_*F*_R_, where *F*_T_ and *F*_R_ are Fourier transform operators with respect to transmitter and receiver coordinates. Then the back-propagation imaging operator can be written in terms of the transmit angular spectrum *G*_T_(*t*, *k*_*x*(*T*)_, *x*_R_) = *F*_T_[*g*_fmc_(*t*, *x*_T_, *x*_R_)] as

3.3 $$\begin{array}{rl} & b(z,x_{T},x_{R})=F_{T}^{-1}F_{R}^{-1}HF_{R}[G_{T}(t,k_{x(T)},x_{R})]\\ \;\mathrm{and}\; & G_{T}(t,k_{x(T)},x_{R})=F_{R}^{-1}H^{-1}F_{R}F_{T}[b(z,x_{T},x_{R})]. \end{array}\}$$

From this expression and using conventional sampling arguments, it follows that the optimal sampling in the wavenumber domain *k*_*x*(*T*)_ is

3.4 $$\Delta k_{x(T)}=\frac{2\pi }{W_{P}},$$

where *W*_*P*_ is the maximum linear size of the PSF in the generalized image domain in the *x*_T_, *x*_R_ directions. It should be noted that, strictly speaking, the size of the PSF in the generalized image domain (points where the PSF is not equal to zero) is unbounded. If the parameter *W*_*P*_ is finite, then this results in additional noise in the image due to aliasing. The level of this noise can be controlled by using some threshold, and then the corresponding value of *W*_*P*_ can be calculated from expressions (2.10) or (2.11), depending on the array size and scatterer location.

### (b) Angular spectrum imaging

One possible practical implementation of the optimal data acquisition approach described above is to transmit angular spectrum components, corresponding to the sampling rule (3.4), and then use all array elements on reception simultaneously similar to the FMC technique. The maximum measured wavenumber is given by $k_{\max}=2\pi /\lambda \sin\varphi_{0}$. Then the number of array firings is given by $2k_{\max}/\Delta k_{x(T)}$, and using (3.4) can be written as

3.5 $$N_{T}=2\sin\varphi_{0}\frac{W_{P}}{\lambda }.$$

Then the image is produced by applying the back-propagation operator in form (3.3). In this case, all information is preserved and the FMC data can be reconstructed from the generalized image using inverse imaging (2.5).

However, there are some difficulties associated with the angular spectrum excitation. Each angular spectrum component represents a one-dimensional wave propagating in the *z*-direction. For high wavenumbers *k*_*x*(*T*)_, this wave is highly dispersive and has very small amplitude compared with the plane wave excitation at *k*_*x*(*T*)_ = 0, which makes it very sensitive to experimental noise. Therefore, a practical implementation of the angular spectrum approach is very challenging.

### (c) Plane wave imaging

**Figure 3.**
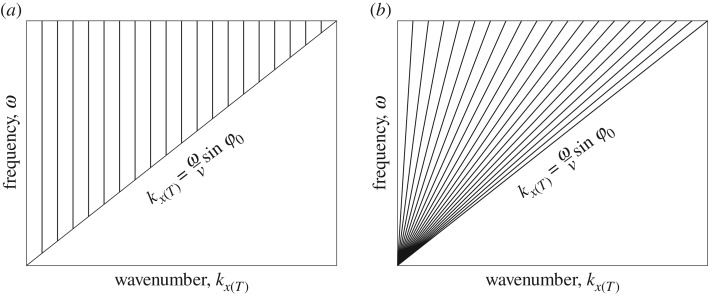
Sampling schemes in wavenumber-frequency, *k*_*x*(*T*)_ − *ω*, domain, corresponding to (*a*) angular spectrum excitation; (*b*) plane wave excitation.

The problems associated with the angular spectrum method can be overcome by using an alternative approach—plane wave imaging, which is described in this section. Figure 3*a* schematically shows the sampling in the wavenumber-frequency domain, *k*_*x*(*T*)_ − *ω*, corresponding to the angular spectrum excitation. Each angular spectrum component is associated with the fixed wavenumber *k*_*x*(*T*)_, which is independent of frequency. It means that the phase velocity of the angular spectrum wave is frequency-dependent. In order to illuminate the dispersion effect the wavenumber *k*_*x*(*T*)_ in the sampling scheme can be taken to be proportional to frequency

3.6 $$k_{x(T)}=\frac{\omega }{v}\sin\varphi .$$

This corresponds to non-uniform sampling and is schematically shown in figure 3*b*. Each component (3.6) represents the plane wave propagating in the direction *φ* relative to the array. Note, that practically each plane wave can be generated by applying appropriate time-delays to the array elements. The reception is performed simultaneously on all array elements as before.

Because the sampling in the wavenumber domain is non-uniform, the back-propagation image cannot be calculated using the Fast Fourier Transform algorithm. Therefore, a numerical evaluation of Fourier integrals on a non-uniform grid must be performed instead. However, a computationally more efficient approach is to use the asymptotic form of the back-propagation method [35]. In this case, the imaging algorithm has a form similar to the total focusing method, and, hence, very efficient numerical implementation is possible. The detailed derivation is given in appendix Ac.

The angular interval, Δ*φ*, of the plane wave excitation can be approximately calculated from expressions (3.4) and (3.6) as

3.7 $$\Delta \varphi =\frac{\lambda }{W_{P}}.$$

For a scatterer located in the centre of the array (*x*_0_ = 0), the PSF is symmetrical in the *x*_T_, *x*_R_ plane. In this case, *W*_*P*_ can be taken as the size of the PSF in the *x*_T_ direction, and can be written as *W*_*P*_ = 2*r*sin*θ*_*P*_ = 2*r*cos0.5*φ*_0_, where *r* is the side-lobe size in the generalized image domain (figure 2*d*). Here relationship (2.7) between size-lobe direction *θ*_*P*_ and the angular array aperture *φ*_0_ was used. Then the angular sampling interval can be expressed as

3.8 $$\Delta \varphi =\frac{1}{2\tilde{r}\cos(\varphi_{0}/2)}.$$

The number of plane waves is *N*_*φ*_ = 2*φ*_0_/Δ*φ*. Finally, using expressions (2.10), (2.11) for asymptotic behaviour of the PSF, it is possible to obtain the following expression for the optimal number of firings:

3.9 $$N_{\varphi }=\min[N_{1\varphi };N_{2\varphi }]$$

and

3.10 $$N_{1\varphi }=\frac{1}{\delta_{P}\pi },\;N_{2\varphi }=\frac{1}{{\left(\delta_{P}\pi \right)}^{2/3}}\frac{1}{{\left(\varphi_{0}\tan(\varphi_{0}/2)\right)}^{1/3}}.$$

Here the variable *δ*_*P*_ describes the relative noise level due to aliasing.

Expression (3.9) allows us to draw some important practical conclusions. Note, that if the location of a scatterer is fixed, then the angle *φ*_0_ monotonically increases when the array aperture increases. Then from (3.9) it follows that for a relatively small angular range *φ*_0_ the required number of firing is constant and independent of *φ*_0_, and, consequently, independent of the array aperture. Moreover, when the number of array elements increases (hence, angular range *φ*_0_ increases), the optimal number of firing decreases as (*φ*_0_tan0.5*φ*_0_)^−1/3^. This behaviour can be explained by the fact that increased angular range results in better focusing, and, hence, smaller size of the PSF.

It should be stressed that the number of transmitted plane waves given by expression (3.9) preserves the PSF in the generalized image domain (to the accuracy given by the relative noise level *δ*_*P*_). It means that in this case the plane wave array data is equivalent to the FMC data and can be converted to the FMC data using inverse imaging. On the other hand, the conventional two-dimensional image corresponds to the pulse-echo *x*_T_ = *x*_R_ plane in the generalized image domain. Therefore, if it is required to reconstruct the two-dimensional image only, then the number of plane wave excitations, *N*_*φ*,image_, is half that given by expression (3.9):

3.11 $$N_{\varphi ,\mathrm{image}}=\frac{1}{2}N_{\varphi }.$$

In this case, the two-dimensional image is preserved (up to the relative noise level *δ*_*P*_), but the part of the generalized image outside of the pulse-echo plane is corrupted by aliasing noise.

It is noted that the results (3.9), (3.11) for the optimal number of plane wave transmissions do not explicitly depend on the position of the scatterer relative to the array and are entirely defined by the angular aperture only. However, it is assumed that the angular aperture is symmetrical, [ − *φ*_0_, *φ*_0_]. In practice, it corresponds to the imaging area directly below the array centre. If a point scatterer is located at the position (*x*_0_, *z*_0_), *x*_0_≠0, that is offset from the array centre, then the angular range, [*φ*_1_, *φ*_2_], is asymmetrical: $\min(|\varphi_{1}|,|\varphi_{2}|)<\varphi_{0}$, $\max(|\varphi_{1}|,|\varphi_{2}|)=\varphi_{0}$. However, the important result obtained above is that the maximum angular step, Δ*φ*, of the plane wave transmissions is inversely proportional to the size of the point spread function, *W*_*P*_, in the generalized image (equation (3.7)). This condition is true for any location of a scatterer, and, therefore, can be used to estimate the minimum number of plane waves. Qualitatively, if a scatterer is located with an offset relative to the array centre, then the focusing performance of the array worsens and the relative side lobe amplitude of the PSF increases. Therefore, for a given relative side lobe level, *δ*_*P*_, the size of the PSF increases. Consequently, the plane wave angular step becomes smaller.

The structure of the PSF in the generalized image domain for an offset scatterer can be investigated similar to the centred scatterer (see appendix Ab). However, the analysis in this case becomes more complex, and, in general, the result cannot be represented in a compact form as in (3.9). Nevertheless, in a particular case of a scatterer located close to the edge of the array, *x*_0_ ≈ *L*/2 (*L* is the array aperture), it is possible to estimate the minimum number of plane waves as (see (3.11))

3.12 $$\frac{1}{2}N_{1}\leq N_{\varphi ,\mathrm{image}}\left[x_{0}=\frac{L}{2}\right]\leq \frac{1}{2}N_{2}$$

and

3.13 $$N_{1}=\min[N_{1\varphi },2^{-1/3}N_{2\varphi }],\;N_{2}=\max[N_{1\varphi },2^{-1/3}N_{2\varphi }],$$

where *N*_1*φ*_, *N*_2*φ*_ are given by (3.10). Note that in this expression it is assumed that the angular range is [0, *φ*_0_], and corresponds to the imaging area around the scatterer location at *x*_0_ = *L*/2. If it is required to perform imaging in the entire area below the array, −0.5*L*≤*x*_0_≤0.5*L*, then the angular range is [ − *φ*_0_, *φ*_0_], and the minimum number of plane waves is given by

3.14 $$N_{1}\leq N_{\varphi ,\mathrm{image}}\left[\frac{L}{2}\leq x_{0}\leq \frac{L}{2}\right]\leq N_{2}.$$

Therefore, in this case the optimal number of array firings is between 2^2/3^ ≈ 1.6 and 2 times larger than for a centred scatterer.

## 4. Experiments

### (a) Scatterer in the centre of the array

**Figure 4.**
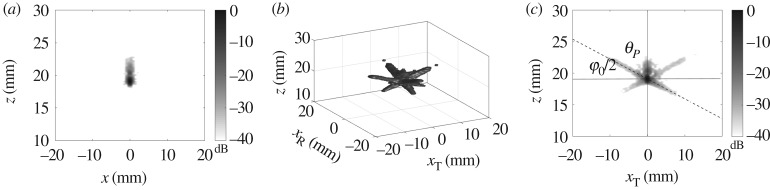
Experimental images for 1 mm side drilled hole obtained from the FMC data: (*a*) back-propagation two-dimensional image; (*b*) generalized back-propagation image, −40 dB isosurfaces; (*c*) slice of the generalized image at *x*_R_ = 0. Dashed line indicates the direction of side-lobes (angle *θ*_*P*_) given by (2.7), angle *φ*_0_ is the array half angular aperture.

The theory developed in the previous sections was experimentally validated on an aluminium specimen with a 1 mm diameter side drilled hole located at 20 mm depth. A linear array with the same parameters (5 MHz, 64 elements, 0.6 mm element pitch) as in the modelling example was used. Firstly, the measurements were performed by positioning the array exactly above the defect. The case of a scatterer located with the offset relative to the array centre is considered later. Figure 4 shows the back-propagation imaging results obtained using the FMC dataset. Note that similar to §2d the half angle filter of 35° was applied in the wavenumber domain. Figure 4*a*,*b* shows the two-dimensional back-propagation image and the generalized image, respectively. Figure 2*d* shows the *x*_T_ − *z* plane of the generalized image at *x*_R_ = 0. The dashed line corresponds to the theoretically predicted side-lobes direction. It can be seen that in general the experimental PSF structure agrees well with the theoretical estimations (see also figure 2*d*).

**Figure 5.**
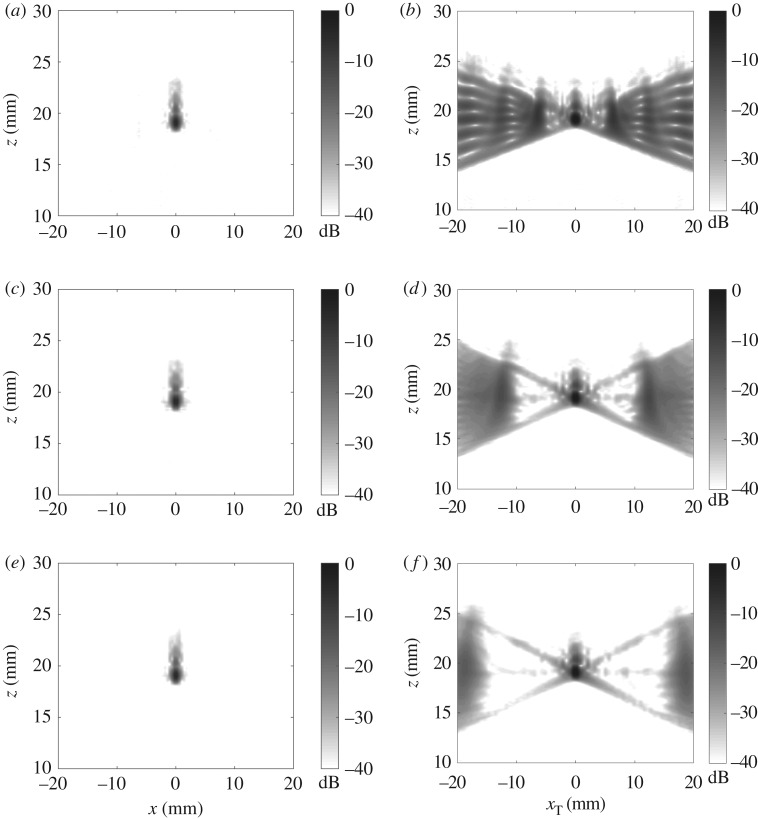
Experimental images for 1 mm side drilled hole obtained with 9 (*a*,*b*), 17 (*c*,*d*) and 25 (*e*,*f*) emitted plane waves and aperture half angle limit of 35°: (*a*,*c*,*e*) back-propagation two-dimensional image; (*b*,*d*,*f*) slice of the generalized image at *x*_R_ = 0.

The plane wave imaging results are presented in figure 5 for 9, 17 and 25 transmitted plane waves. The maximum steering angle is 35° and the angular filter of 35° is also applied on reception in the wavenumber domain. Note that according to expression (3.11) only nine plane waves are required for the maximum noise level of −40 dB in the two-dimensional image. It can be seen that in all cases the two-dimensional images are practically identical and the noise level is below −40 dB (see also figure 4*a* for the two-dimensional image obtained from the FMC data).

Figure 5*b*,*d*,*f* illustrates how the aliasing noise in the generalized image domain changes with the number of emitted plane waves. For example, if only nine plane waves are used then the generalized image is completely corrupted with noise in the region outside the pulse-echo plane |*x*_T_ − *x*_R_|≥2 mm. However, if the number of emissions is increased to 17 plane waves, then the aliasing noise in the generalized image domain does not affect the data as predicted by expression (3.9).

**Figure 6.**
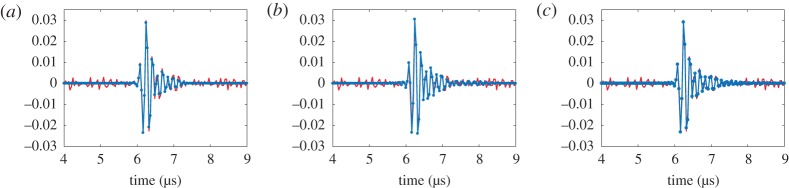
Time trace corresponding to transmitter element 40 and receiver element 20 (red lines) and the same time trace reconstructed using the inverse imaging method from the plane wave dataset with (*a*) 9, (*b*) 17 and (*c*) 25 emissions (blue lines).

**Figure 7.**
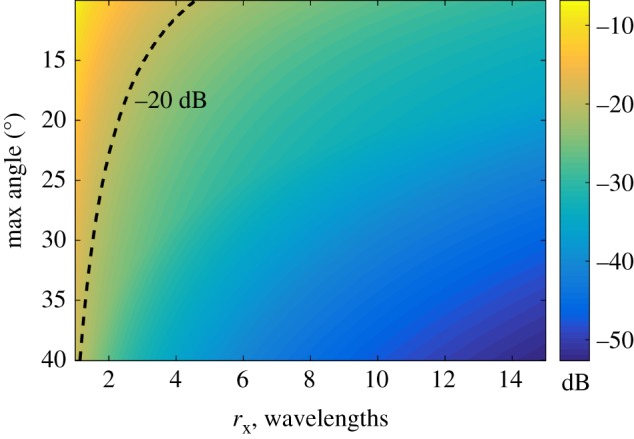
Relative side lobe amplitude of the PSF in the generalized image domain as a function of distance *r*_*x*_ in the *x*_T,R_ direction and angular aperture. (Online version in colour.)

Once the generalized image is constructed, it can be converted to the FMC data using the inverse imaging process. Preliminarily, the part of the generalized image free from the aliasing noise was filtered out by applying the spatial filter |*x*_*T*,*R*_|≤Δ*x*, *z*_1_≤*z*≤*z*_2_. Based on figure 5, it can be determined that *z*_1_ = 18 mm, *z*_2_ = 23 mm and Δ*x* = 2, 8 and 10 mm for 9, 17 and 25 plane wave emissions, respectively. An example of the reconstructed time-trace corresponding to transmitter element 40 and receiver element 20 is shown in figure 6, where for comparison the same time-trace from the experimentally captured FMC dataset is also shown. It can be seen that in all three cases the reconstruction is very good, even in the case of only nine plane wave transmissions. This surprising result is explained by noting that the majority of the PSF data in the generalized image domain, which is needed for the inverse imaging, is concentrated in a relatively small area around the defect location. In order to quantitatively illustrate this fact the relative side lobe amplitude of the PSF as a function of array half angular aperture, *φ*_0_, and the distance in *x*_T,R_ direction is shown in figure 7. The calculations were performed using expression

4.1 $$\delta_{P}=\min[\delta_{P1},\delta_{P2}].$$

Here *δ*_*P*1_, *δ*_*P*2_ are given by formulae (2.10) and (2.11) and the distance $\tilde{r}$ in the side lobe direction was replaced by $\tilde{r}={\tilde{r}}_{x}/\cos(\varphi_{0}/2)$, where ${\tilde{r}}_{x}$ is the distance in *x*_T,R_ direction. For example, it is seen that for *φ*_0_ = 35° the PSF data with a relative amplitude above −20 dB is contained in the region |*x*_*T*,*R*_|≤1.4*λ*, where the ultrasonic wavelength *λ* = 1.2 mm at 5 MHz.

**Figure 8.**
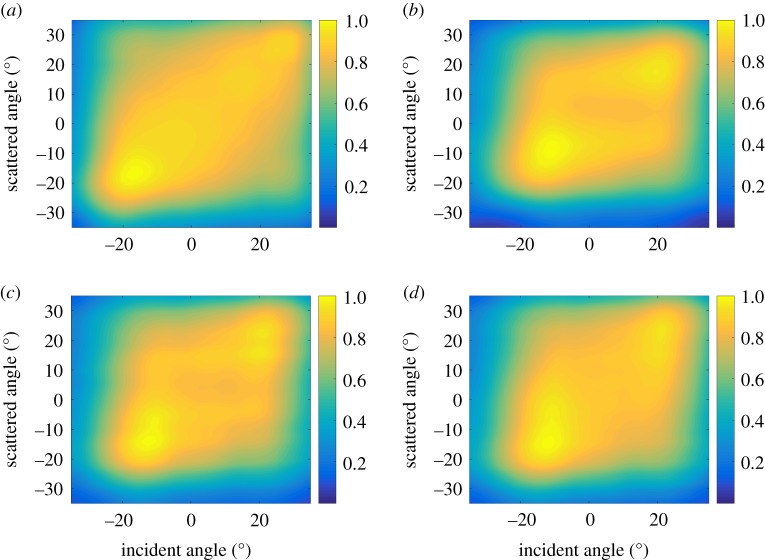
Experimental scattering matrices for 1 mm diameter hole extracted from the FMC data (*a*) and the plane wave data with (*b*) 9, (*c*) 17 and (*d*) 25 emissions and aperture half angle limit of 35°. Scattering matrices are normalized to its maximum values. (Online version in colour.)

The possibility to reconstruct the FMC dataset means that all signal processing operations available for the FMC data can also be applied to the plane wave dataset. For example, the scattering matrices extracted from the FMC data and the plane wave data with 9, 17 and 25 emissions are shown in figure 8. For the plane wave data, the FMC data were reconstructed first using the inverse imaging as described above and then the scattering matrix was extracted from the FMC data. It is seen that the results are very similar confirming that the number of plane waves given by expression (3.11) is enough to preserve the majority of FMC information. Note that the scattering matrices extracted from the FMC data and nine plane wave data (figure 8*a*,*b*) differ mainly at the maximum angles ±*φ*_0_ = ±35°. This is consistent with the asymptotic analysis performed in appendix Ab, where it has been shown that the main side lobe contribution is given by the maximum steering angles and the stationary point (0°). However, this information is partly removed from the plane wave data during the preliminary filtering in the generalized image domain before the application of the inverse imaging operator.

**Figure 9.**
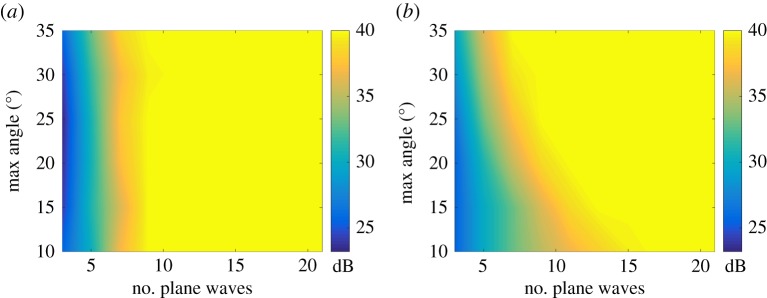
Signal-to-noise ratio in the two-dimensional image as a function of the number of plane waves and angular aperture. (*a*) Experimental results for 1 mm diameter hole; (*b*) Theoretical predictions (expressions (3.9) and (3.11)).

Finally, the signal-to-noise ratio (SNR) in the two-dimensional image as a function of number of plane waves and angular aperture was calculated. The noise amplitude was estimated as the maximum image amplitude in the area outside the rectangular box containing the PSF. The half *x*-size of the box was taken equal to $2\pi /\max k_{x(T)}=\lambda /\sin\varphi_{0}$, and the size in the *z*-direction was 6 mm. The result is presented in figure 9*a*. Also figure 9*b* shows the SNR based on the theoretical formulae (3.9) and (3.11). It can be seen that there is a reasonable agreement, however, for angular apertures smaller than 20° the experimental images show slightly higher SNR compared with the theoretical predictions. This can be explained by the fact that asymptotic expression (3.9) is based on the assumption of regular sampling in the wavenumber domain. In this case, the PSF pattern is periodically repeated in the *x*_T_ direction. Plane wave imaging however corresponds to irregular sampling, which is also frequency-dependent (figure 3). Consequently, the grating lobe structure in the generalized image domain is different (figure 5). It is important to note that this modelling approach therefore represents a good conservative approach.

### (b) Scatterer offset from the array centre

In this section, a scatterer located with an offset to the array centre is considered. In order to illustrate the plane wave imaging performance in this case a modelling example is presented first. The linear array aperture for the array parameters given in §2a is *L* = 37.8 mm, the point scatterer is located at a depth of *z*_0_ = 20 mm and its lateral position *x*_0_ varies from 0 (array centre) to *x*_0_ = *L*/2 (array edge). The maximum plane wave steering angle is *φ*_0_ = 35° as in the case of the centred scatterer. Therefore, the angular aperture of the scatterer is [ − *φ*_*x*_, *φ*_0_], 0≤*φ*_*x*_≤*φ*_0_, and *φ*_*x*_ = 0° corresponds to the scatterer located at the array edge and *φ*_*x*_ = *φ*_0_ corresponds to the scatterer at the array centre.

**Figure 10.**
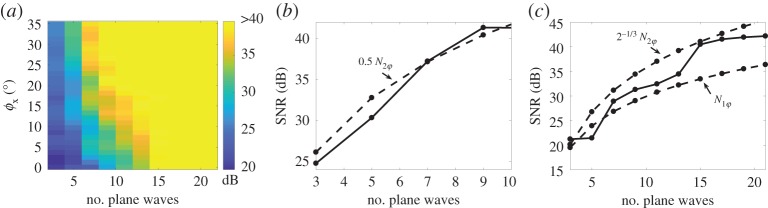
(*a*) Modelling results of the signal-to-noise ratio in the two-dimensional image as a function of the number of plane waves and lateral position of the scatterer. The scatterer is located at the depth of *z*_0_ = 20 below the array, the maximum plane wave steering angle is *φ*_0_ = 35°. Angular aperture of the scatterer is [ − *φ*_*x*_, *φ*_0_], 0≤*φ*_*x*_≤*φ*_0_. The number of plane waves corresponds to the full angular aperture [ − *φ*_0_, *φ*_0_]. (*b*) The case *φ*_*x*_ = *φ*_0_ for the scatterer at the array centre; (*c*) the case *φ*_*x*_ = 0° for the scatterer located at the array edge. Dashed lines in (*b*) and (*c*) correspond to theoretical prediction (3.14).

Initially, the FMC array data were modelled using the far-field ray tracing model [11,12]. Then the FMC data were converted into the plane wave array data by applying appropriate time-delays to the array elements on transmission. Figure 10*a* shows the signal to aliasing noise ratio in the conventional two-dimensional image as a function of the number of emitted plane waves and the scatterer location. Similar to the previous section, the noise amplitude was estimated as the maximum image amplitude in the area outside the rectangular box containing the peak of the PSF. The size of the box was chosen to satisfy the condition that the amplitude of the PSF in the back-propagation image (FMC dataset) is less than −40 dB in the area away from the selected region.

It is important to note, that in figure 10*a* the number of plane waves for a given angle *φ*_*x*_ is related to the full angular aperture [ − 35°, 35°], and, consequently, corresponds to the case when the scatterers are located in the whole area −*x*_0_≤*x*≤*x*_0_. It can be seen that for a given number of plane waves the relative level of aliasing noise gradually increases as the position of the scatterer approaches the edge of the array, *φ*_*x*_ → 0. Additionally, figure 10*b*,*c* separately shows the SNR for the centred scatterer and the scatterer located at the array edge together with the theoretical predictions (3.11) and (3.14). It can be seen that the agreement is very good. In particular, the number of plane waves required to image the entire area below the array, −*L*/2≤*x*_0_≤*L*/2, with the SNR greater than 40 dB is 2^−1/3^*N*_2*φ*_ ≈ 15 as predicted by the lower bound of the theoretical condition (3.14).

**Figure 11.**
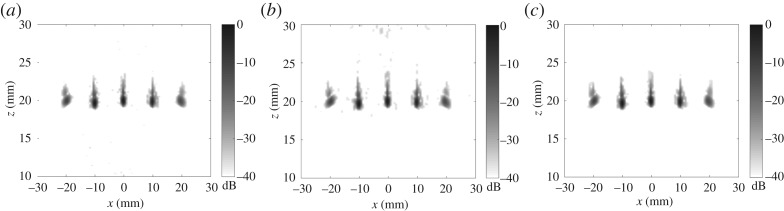
Experimental images for 1 mm side drilled holes with a pitch of 10 mm at a depth of 20 mm: (*a*) back-propagation image obtained from the FMC array data; (*b*) plane wave image with nine transmitted plane waves; (*c*) plane wave image with 15 transmitted plane waves. In all cases, the angular aperture on transmission and reception is [ − 35°, 35°].

So far only a single scatterer case has been considered. However, because imaging operations are linear, all results remain valid even when more than one scatterer is present, providing that the single scattering contribution is dominant. This is supported by the experimental measurements shown in figure 11. In this case, an aluminium specimen was used with a row of 1 mm diameter side drilled holes with a pitch of 10 mm at a depth of 20 mm. Figure 11*a* shows the back-propagation imaging results obtained using the FMC dataset. The half angle filter of 35° was applied in the wavenumber domain. The plane wave images for 9 and 15 transmitted plane waves are presented in figure 11*b*,*c*. The steering angle is [ − 35°, 35°] and the angular filter of 35° was also applied on reception. The aliasing noise is visible in the case of nine plane waves predominantly between and above the scatterers. However, the noise amplitude is at approximately −36 dB, which is half that predicted by the theoretical estimation of −30 dB (figure 10*c*). This is explained by the fact that the highest aliasing noise is related to the side scatterers, but their absolute amplitude is smaller compared with the scatterer at the array centre due to the reduced angular range. In the case of 15 plane waves, it can be seen that the image is practically identical to the back-propagation image and the noise level is below −40 dB as predicted theoretically.

### (c) Other factors affecting plane wave imaging

One of the main results of this paper is the fact that the optimum number of plane waves is determined by the array PSF in the generalized image domain. Therefore, the structure of the PSF can be used to assess the influence of different parameters on the plane wave imaging performance. As an example, two factors which are important from the practical point of view are briefly considered in this section: array element pitch and multiple scattering. Note, that only a qualitative illustration is given below, and the detailed quantitative analysis of these cases will be the subject of future work.

**Figure 12.**
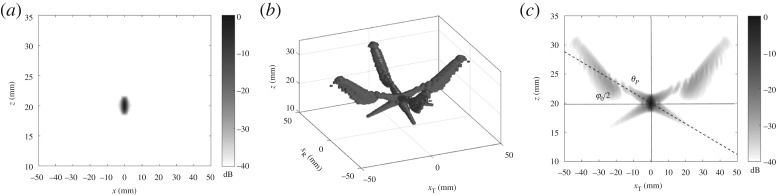
Point spread function for the 5 MHz, 64 element array with 1*λ* element pitch and angular filter of *φ*_0_ = 20°: (*a*) back-propagation two-dimensional image; (*b*) generalized back-propagation image, −40 dB isosurfaces; (*c*) slice of the generalized image at *x*_R_ = 0. Dashed line indicates the direction of side-lobes (angle *θ*_*P*_) given by (2.7).

The developed theory implicitly assumes that the array element pitch satisfies to the Nyquist sampling criterion (*λ*/2). However in many fields, including medical ultrasonic imaging it is common to use arrays with *λ*-pitch. Figure 12 shows the modelling example of a conventional two-dimensional image and the generalized image of the PSF for the same 5 MHz 64 element array as in §2, but with *λ* element pitch. Additionally, an angular filter with the half angular aperture of 20° was applied on transmission and reception [36]. It can be seen that grating lobes are successfully suppressed in the two-dimensional image. However, there are significant grating lobes in the generalized image domain outside the pulse-echo plane. These grating lobes do not appear on the two-dimensional image, but effectively result in an increased size of the PSF, and, therefore, affect the minimum number of array firings required to preserve all information. This provides the quantitative framework to explain why images can look fine with a reduced number of firings and specifies the number required to achieve this, something previously not reported.

Another important aspect is related to the single scattering assumption. Strictly speaking, a multiple scattering contribution always exists, although in many cases it is small compared with single scattering [37]. In the generalized image, domain multiple scattering results in additional noise distributed outside the conventional two-dimensional image plane *x*_T_ = *x*_R_. This ‘multiple scattering’ part of the generalized image will result in aliasing noise in the two-dimensional image during the plane wave imaging with reduced number of firings. Essentially, this will degrade the performance of the two-dimensional image and the magnitude and nature of this effect must be taken into account when designing the reduced plane wave firings.

## 5. Conclusion

The problem of optimization of ultrasonic array data acquisition has been investigated. The conventional FMC technique involves collecting all possible transmitter–receiver signals and, therefore, contains the maximum information which can be measured by an array. However, the volume of measured data is very large and the data acquisition is not fast enough for some applications.

It has been shown that under the single scattering assumption the FMC dataset has a specific sparse structure. This property can be used to design an optimal data acquisition method. Two possible strategies, corresponding to different sampling schemes in the wavenumber-frequency domain, have been considered: the angular spectrum and plane wave imaging techniques. The analytical relationship between the number of emissions, angular array aperture and signal-to-noise ratio has been derived. An important counterintuitive conclusion from this relationship, is that the optimal number of emissions decreases when the angular aperture of the array increases.

The analysis shows that plane wave imaging is more suitable for practical implementation than the angular spectrum method. The theory has been validated experimentally using 5 MHz, 64 element array measurements on an aluminium sample with a 1 mm diameter holes. In particular, it has been demonstrated that the theoretical prediction of the optimal number of plane waves is in a good agreement with the experiment. Also, it has been shown that the FMC dataset can be reconstructed from the plane wave data with a reduced number of emissions. This result means that all signal processing operations available for the FMC data can also be applied to the plane wave dataset. For example, the scattering matrices of the defects can be extracted and used for defect characterization.

The possible applications of the developed theory are twofold. Firstly, the precise knowledge of the PSF structure gives an opportunity to construct an efficient spatial filter in the generalized image domain for the accurate extraction of defect scattering matrices. Secondly, the optimal number of array firings for various practical configurations can be predicted and used for fast imaging without loss of scattering information, or with detailed knowledge of what information has been discarded.

## Supplementary Material

Experimental array data, 1 mm hole at 20 mm depth, Full Matrix Capture

## Supplementary Material

Experimental array data, 1 mm hole at 20 mm depth, 9 plane waves

## Supplementary Material

Experimental array data, 1 mm hole at 20 mm depth, 17 plane waves

## Supplementary Material

Experimental array data, 1mm hole at 20 mm depth, 25 plane waves

## Figures and Tables

**Figure A.1.**
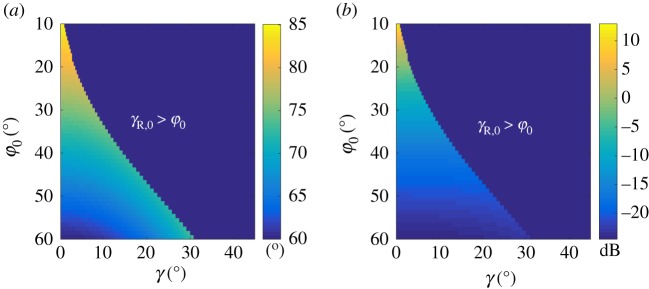
(*a*) Solution to equation (A 12) in the form *θ*(*γ*, *φ*_0_) in the case *m* = 0, *n* = 2; (*b*) relative side lobe amplitude (expression A 13) at *r* = *λ* as a function of azimuth *γ* in the generalized image domain and half angular aperture *φ*_0_. (Online version in colour.)

## Data accessibility

The datasets supporting this article have been uploaded as part of the electronic supplementary material.

## Appendix A

**(a) Back-propagation of angular spectrum operator**

In this section, the expression for the back-propagation of angular spectrum operator *H* is given. Any time-domain signal *u*(*t*) can be expressed as a linear superposition of its spectral components *u*(*ω*)

A 1 $$u(t)=\frac{1}{2\pi }\int u(\omega )\;e^{i\omega t}\;d\omega .$$

The Fourier transform operator *F* transforms the FMC array data *g*_fmc_(*t*, *x*_T_, *x*_R_) into the angular spectrum *G*(*t*, *k*_*x*(*T*)_, *k*_*x*(*R*)_), where *k*_*x*(*T*)_ and *k*_*x*(*R*)_ are the wavenumbers in the *x*-direction for the transmitted and scattered waves, respectively:

A 2 $$\begin{array}{rl} & G(t,k_{x(T)},k_{x(R)})\equiv F[g_{\mathrm{fmc}}(t,x_{T},x_{R})]\\ & \;=\iint g_{\mathrm{fmc}}(t,x_{T},x_{R})\;e^{i(k_{x(T)}x_{T}+k_{x(R)}x_{R})}\;dx_{T}\;dx_{R}. \end{array}$$

Note, that the back-propagation operation is based on the assumption that the transmitter and receiver elements are sensitive to the longitudinal wave mode only. Then it can be shown [12] that the angular spectrum, *G*(*t*, *k*_*x*(*T*)_, *k*_*x*(*R*)_), represents a one-dimensional wave propagating in the *z*-direction with the wavenumber *k*_*z*_ = *k*_*z*(*T*)_ + *k*_*z*(*R*)_. Here $k_{z(T)}=\sqrt{k^{2}-k_{x(T)}}$, $k_{z(R)}=\sqrt{k^{2}-k_{x(R)}}$ are the wavenumbers in the *z*-direction for the transmitted and scattered wave, and *k* = *ω*/*v* is the scalar wavenumber, where *v* is the velocity of the longitudinal wave. The back-propagation of the angular spectrum operator *H* converts the time data *G*(*t*, *k*_*x*(*T*)_, *k*_*x*(*R*)_) into a function of propagation distance, *h*(*z*, *k*_*x*(*T*)_, *k*_*x*(*R*)_), and can be written in the form:

A 3 $$\begin{array}{rl} & h(z,k_{x(T)},k_{x(R)})\equiv H[G(t,k_{x(T)},k_{x(R)})]\\ & \;=\frac{1}{2\pi }\int G(\omega ,k_{x(T)},k_{x(R)})\;e^{ik_{z}z}\;d\omega . \end{array}$$

Note that for the spatial wavenumbers *k*_*x*(*T*)_, *k*_*x*(*R*)_ > *k* the spectrum *G*(*ω*, *k*_*x*(*T*)_, *k*_*x*(*R*)_) corresponds to the exponentially decaying evanescent waves and it can be assumed that *G*(*ω*, *k*_*x*(*T*)_, *k*_*x*(*R*)_) = 0 for *k*_*x*(*T*)_, *k*_*x*(*R*)_ > *k*. If *k*_*x*(*T*)_≠0 or *k*_*x*(*R*)_≠0 the one-dimensional wave *G*(*t*, *k*_*x*(*T*)_, *k*_*x*(*R*)_) is dispersive and the back-propagation of the angular spectrum (A 3) is equivalent to the dispersion compensation method [38]. The wavenumber *k*_*z*_ nonlinearly depends on the frequency *ω* and direct calculation using the formula (A 3) is very time consuming. However, if the integration variable is changed from *ω* to *k*_*z*_ then the integral (A 3) can be written in the form of a Fourier transform with respect to the variable *k*_*z*_ as [38]

A 4 $$h(z,k_{x(T)},k_{x(R)})=\frac{1}{2\pi }\int G(\omega (k_{z}),k_{x(T)},k_{x(R)}){\left(\frac{dk_{z}}{d\omega }\right)}^{-1}\;e^{ik_{z}z}\;dk_{z}.$$

**(b) Asymptotic analysis of the array point spread function in the generalized image domain**

In this section, the asymptotic analysis of the array PSF in the generalized image domain, (*x*_T_, *x*_R_, *z*), is performed. The point scatterer is located at the depth *z*_0_ directly below the array centre (*x*_0_ = 0). It is convenient to study the structure of the PSF in the spherical coordinate system, (*r*, *θ*, *γ*), with the centre at the scatterer location:

A 5 $$x_{T}=r\sin\theta \cos\gamma ,\;x_{R}=r\sin\theta \sin\gamma \;\mathrm{and}\;z-z_{0}=r\cos\theta .$$

It is also useful to change the wavenumber variables in the Fourier integrals in the back-propagation operator (3.1) as *k*_*x*(*T*,*R*)_ =  − *k*sin*γ*_T,R_. Here angles −*φ*_0_≤*γ*_T,R_≤*φ*_0_ and *φ*_0_ is half of the array angular aperture. Then using the model of array data developed in [12], the envelope of the generalized image, *b*(*r*, *θ*, *γ*), can be expressed as

A 6 $$\begin{array}{rl} b(r,\theta ,\gamma ) & =\left|\int_{\omega_{0}-\Delta \omega }^{\omega_{0}+\Delta \omega }U_{0}(\omega )I_{\mathrm{TR}}(\omega ,r,\theta ,\gamma )\;d\omega \right|\\ \;\mathrm{and}\;I_{\mathrm{TR}} & =\int_{-\varphi_{0}}^{\varphi_{0}}\int_{-\varphi_{0}}^{\varphi_{0}}f(\gamma_{T})S(\gamma_{T},\gamma_{R})f(\gamma_{R})\;e^{ikr(\Phi_{T}+\Phi_{R})}\;d\gamma_{T}\;d\gamma_{R}, \end{array}\}$$

where *U*_0_(*ω*) is the frequency spectrum of the initial pulse *u*_0_(*t*) with the centre frequency *ω*_0_ and half-bandwidth Δ*ω*. Below it is assumed that $\max|u_{0}(t)|=u_{0}(0)=1$. The function *f*(*γ*) is the array element directivity function, *S*(*γ*_T_, *γ*_R_) is the scattering matrix, and the phase factors *Φ*_T,R_ are given by

A 7 $$\begin{array}{rl} \Phi_{T} & =\sin\theta \cos\gamma \sin\gamma_{T}+\cos\theta \cos\gamma_{T}\\ \;\mathrm{and}\;\Phi_{R} & =\sin\theta \sin\gamma \sin\gamma_{R}+\cos\theta \cos\gamma_{R}. \end{array}\}$$

It is assumed that the scatterer and array elements are omnidirectional, so *f*≡1 and *S*≡1. In this case,

A 8 $$I_{\mathrm{TR}}=I_{T}I_{R},\;I_{T,R}=\int_{-\varphi_{0}}^{\varphi_{0}}e^{ikr\Phi_{T,R}}\;d\gamma_{T,R}.$$

Integrals *I*_T,R_ can be evaluated using the stationary phase method:

A 9 $$\begin{array}{rl} I & \approx \frac{1}{\sqrt{r}}I_{0}\;e^{ikr\Phi_{0}}+\frac{1}{r}(I_{1}\;e^{ikr\Phi_{1}}-I_{2}\;e^{ikr\Phi_{2}}),\\ \Phi_{0} & =\Phi (\gamma_{0}),\;\Phi_{1}=\Phi (\varphi_{0}),\;\Phi_{2}=\Phi (-\varphi_{0})\\ \;\mathrm{and}\;I_{0} & =\sqrt{\frac{2\pi }{k|\Phi^{\prime \prime }(\gamma_{0})|}}\;e^{i\pi /4\;\mathrm{sgn}\;\Phi^{\prime \prime }(\gamma_{0})},\;I_{1}=\frac{1}{ik\Phi^{\prime }(\varphi_{0})},\;I_{2}=\frac{1}{ik\Phi^{\prime }(-\varphi_{0})}. \end{array}\}$$

Here subscripts *T*, *R* are omitted to simplify notations. The term *I*_0_ represents the contribution of the stationary point *γ*_0_, *Φ*′(*γ*_0_) = 0. The other two terms, *I*_1_ and *I*_2_, correspond to the boundary points ±*φ*_0_.

From (A 6) and (A 9), it follows that the generalized image can be expressed as:

A 10 $$\begin{array}{rl} b & =\left|\sum_{n,m=0}^{2}b_{nm}\right|\\ \;\mathrm{and}\;b_{nm} & =\frac{1}{r^{\alpha }}\int_{\omega_{0}-\Delta \omega }^{\omega_{0}+\Delta \omega }U_{0}(\omega )I_{T,n}(\omega )I_{R,m}(\omega )\;e^{i\omega r/v(\Phi_{T,n}+\Phi_{R,m})}\;d\omega , \end{array}\}$$

where parameter *α* = 1, 1.5, 2 depending on the *n* and *m*. These integrals can be approximately calculated as

A 11 $$|b_{nm}|\approx \frac{2\pi }{r^{\alpha }}|I_{T,n}(\omega_{0})I_{R,m}(\omega_{0})|u_{0h}\left(\frac{r}{v}(\Phi_{T,n}+\Phi_{R,m})\right),$$

where *u*_0*h*_(*t*) is the envelope of the initial pulse *u*_0_(*t*). Because the signal *u*_0_(*t*) has finite duration, from (A 11) it can be concluded that *b*_*nm*_ ≈ 0 as *r* → ∞. The maximum side lobe amplitude is reached in the directions (*γ*, *θ*) where

A 12 $$\Phi_{T,n}(\gamma ,\theta )+\Phi_{R,m}(\gamma ,\theta )\equiv 0.$$

Because the PSF is symmetrical, it is enough to consider directions 0≤*γ*≤*π*/4, 0≤*θ*≤*π*/2. Numerical calculations show that in this case condition (A 12) is satisfied only for *m* = 0 (stationary point in the integral *I*_R_) and *n* = 2 (boundary point in the integral *I*_T_), or *m* = 1, 2 and *n* = 1, 2 (boundary points in the integrals *I*_T_ and *I*_R_). Therefore, from (A 9) it follows that the leading term in the asymptotic expansion of side lobes corresponds to *m* = 0, *n* = 2 and decays as *r*^−3/2^. Note that the maximum value of the PSF corresponds to the scatterer location *r* = 0, and equal to $\max|b|=8\pi \varphi_{0}^{2}$. Then the relative side lobe level, $\delta_{P}=|b|/\max|b|$, can be written as

A 13 $$\delta_{P}={\left(\frac{\lambda }{r}\right)}^{3/2}\frac{1}{8\pi \varphi_{0}^{2}}{\left|\frac{\partial^{2}\Phi_{R}}{\partial \gamma_{R}^{2}}\right|}_{\gamma_{R}=\gamma_{R,0}}^{-1/2}{\left|\frac{\partial \Phi_{T}}{\partial \gamma_{T}}\right|}_{\gamma_{T}=-\varphi_{0}}^{-1},$$

where *γ*_R, 0_ is the stationary point for the phase function *Φ*_R_, and angles (*γ*, *θ*) in the expressions for the phase functions *Φ*_T,R_ are solutions to equation (A 12) for *m* = 0, *n* = 2.

Equation (A 12) can be solved with respect to the elevation angle *θ* for each value of azimuth *γ* and half angular aperture *φ*_0_, and the solution, *θ*(*γ*, *φ*_0_), for the case *m* = 0, *n* = 2 is shown in figure 13*a*. Then this function *θ*(*γ*, *φ*_0_) is used to calculate the relative side lobe level (A 13) and the result is shown in figure 13*b*. Note that if the stationary point is located outside the array aperture, *γ*_0_ > *φ*_0_, then the main contribution to the side lobe amplitude is given by the boundary points and the leading term in the asymptotic expansion is *O*(*r*^−2^). From figure 13, it is seen that dependance on the azimuth angle is very weak and the side lobes are located predominantly in *γ* = 0 direction (*x*_T_ − *z* plane of the generalized image).

In the case of *γ* = 0, the phase function in the integral *I*_R_ is *Φ*_ R_ = cos*θ*cos*γ*_R_ and the corresponding stationary point is *γ*_ R, 0_ = 0. Condition (A 12) can be written as

A 14 $$(1+\cos\varphi_{0})\cos\theta_{P}\pm \sin\theta_{P}\sin\varphi_{0}=0.$$

This equation allows analytical solution *θ*_*P*_ = *π*/2∓*φ*_0_/2. Then the relative side lobe amplitude can be obtained by substituting *γ* = 0, *θ* = *θ*_*P*_ into formula (A 13), which results in expression (2.11).

It should be noted that the stationary phase approximation of the integral *I*_R_ is based on the Taylor series of the phase *Φ*_R_ around the stationary point *γ*_R, 0_ = 0: *Φ*_ R_ ≈ (1 − *γ*^2^_R_/2)cos*θ*. However, if the angular aperture, *φ*_0_, is small, so *krφ*^2^_0_cos*θ*_*P*_ ≈ 0.5 *kr* *φ*^3^_0_≪2*π*, then the phase *Φ*_R_ ≈ *Φ*_R_(*γ*_R, 0_) can be considered constant over the integration interval and

A 15 $$I_{R}\approx 2\varphi_{0}\;e^{ikr\Phi_{R}(\gamma_{R,0})}.$$

The relative side lobe level in this case can be calculated as previously and is given by formula (2.10).

**(c) Asymptotic form of the back-propagation imaging method**

This section derives an alternative expression for the back-propagation imaging method. This expression specifically takes into account the plane wave data acquisition method and provides a more efficient numerical implementation than expression (3.3).

As has been shown before, the back-propagation imaging operator can be written in the form given in (3.3). By using expression (A 3) for the back-propagation of the angular spectrum operator and rearranging the integration, the back-propagation operator can be expressed as

A 16 $$b(z,x_{T}^{\prime },x_{R}^{\prime })=\frac{1}{8\pi^{3}}\int \left[g_{\mathrm{pw}}(\omega ,k_{x(T)},x_{R})\left\{\int e^{ik_{R}(r_{R}-r_{R}^{\prime })}\;dk_{x(R)}\right\}e^{ik_{T}r_{T}^{\prime }}\;\right]d\omega \;dk_{x(T)}\;dx_{R}.$$

Here *g*_pw_ is the plane wave data,

A 17 $$g_{\mathrm{pw}}=F_{T}[g_{\mathrm{fmc}}],$$

and vectors **r**_(*T*,*R*)_, **r**′_(*T*,*R*)_, **k**_(*T*,*R*)_ are defined as

A 18 $$\begin{array}{rl} r_{T} & ={\left\{x_{T},0\right\}}^{T},\;r_{R}={\left\{x_{R},0\right\}}^{T},\\ r_{T}^{\prime } & ={\left\{x_{T}^{\prime },z\right\}}^{T},\;r_{R}^{\prime }={\left\{x_{R}^{\prime },z\right\}}^{T}\\ \;\mathrm{and}\;k_{T} & ={\left\{k_{x(T)},k_{z(T)}\right\}}^{T},\;k_{R}={\left\{k_{x(R)},-k_{z(R)}\right\}}^{T}. \end{array}\}$$

The integral in the curly brackets can be evaluated explicitly as

A 19 $$\int e^{ik_{R}(r_{R}-r_{R}^{\prime })}\;dk_{x(R)}=i\pi \frac{\partial }{\partial z}H_{0}^{\left(1\right)}(kR_{R}),$$

where *R*_R_ = |**r**_R_ − **r**′_R_|.

Finally, using the far-field asymptotic approximation for the Hankel function *H*^(1)^_0_, the back-propagation operator can be written in the form

A 20 $$B[g_{tfm}(t,x_{T},x_{R})]=\frac{1}{2\pi }\int s(\omega ,x_{T},x_{R},x_{T}^{\prime },x_{R}^{\prime },z)g_{\mathrm{pw}}(\omega ,\gamma_{T},x_{R})\;d\omega \;d\gamma_{T}\;dx_{R},$$

where the angle *γ*_T_ defines the direction of the transmitted plane wave, and *γ*_ T_ = 0 corresponds to the positive *z*-direction. The function *s* represents focusing coefficients

A 21 $$\begin{array}{rl} s & ={\left(\frac{ik}{2\pi }\right)}^{3/2}\frac{\cos\gamma_{T}\cos\gamma_{R}}{\sqrt{R_{R}}}s_{\mathrm{pw}}\\ \;\mathrm{and}\;s_{\mathrm{pw}} & =e^{ik_{T}r_{T}}\;e^{ikR_{R}}, \end{array}\}$$

here the angle *γ*_R_ defines the direction of the vector **r**′_R_ − **r**_R_, so cos*γ*_R_ = *z*/|**r**′_R_ − **r**_R_|. Note, that focusing coefficients *s*_pw_ correspond to the plane wave compounding method [30].
